# Tumor necrosis factor receptor 2-signaling in CD133-expressing cells in renal clear cell carcinoma

**DOI:** 10.18632/oncotarget.8125

**Published:** 2016-03-16

**Authors:** Rafia S Al-Lamki, Jun Wang, Jun Yang, Natalie Burrows, Patrick H Maxwell, Timothy Eisen, Anne Y Warren, Sakari Vanharanta, Simon Pacey, Peter Vandenabeele, Jordan S Pober, John R Bradley

**Affiliations:** ^1^Department of Medicine, NIHR Cambridge Biomedical Research Centre, University of Cambridge, Cambridge CB2 0QQ, UK; ^2^School of Clinical Medicine, Cambridge Institute of Medical Research, University of Cambridge, Cambridge Biomedical Campus, Cambridge CB2 0XY, UK; ^3^Department of Oncology, University of Cambridge, Cambridge CB2 0QQ, UK; ^4^Department of Pathology, Addenbrooke's Hospital, Cambridge CB2 0QQ, UK; ^5^MRC Cancer Unit, University of Cambridge, Hutchison/MRC Research Centre, Cambridge CB2 0XZ, UK; ^6^VIB Inflammation Research Center, Ghent University, UGhent-VIB Research Building FSVM, 9052 Ghent, Belgium; ^7^Department of Immunobiology, Yale University School of Medicine, New Haven, Connecticut 06520-8089, USA

**Keywords:** renal clear cell carcinoma, TNFR2, CD133, cyclophosphamide

## Abstract

Compared to normal kidney, renal clear cell carcinomas (ccRCC) contain increased numbers of interstitial, non-hematopoietic CD133^+^cells that express stem cell markers and exhibit low rates of proliferation. These cells fail to form tumors upon transplantation but support tumor formation by differentiated malignant cells. We hypothesized that killing of ccRCC CD133^+^ (RCC^CD133+^) cells by cytotoxic agents might be enhanced by inducing them to divide. Since tumor necrosis factor-alpha (TNF), signalling through TNFR2, induces proliferation of malignant renal tubular epithelial cells, we investigated whether TNFR2 might similarly affect RCC^CD133+^cells. We compared treating organ cultures of ccRCC *vs* adjacent nontumour kidney (NK) and RCC^CD133+^ *vs* NK CD133^+^ (NK^CD133+^) cell cultures with wild-type TNF (wtTNF) or TNF muteins selective for TNFR1 (R1TNF) or TNFR2 (R2TNF). In organ cultures, R2TNF increased expression of TNFR2 and promoted cell cycle entry of both RCC^CD133+^ and NK^CD133+^ but effects were greater in RCC^CD133+^. In contrast, R1TNF increased TNFR1 expression and promoted cell death. Importantly, cyclophosphamide triggered much more cell death in RCC^CD133+^ and NK^CD133+^cells pre-treated with R2TNF as compared to untreated controls. We conclude that selective engagement of TNFR2 by TNF can drives RCC^CD133+^ proliferation and thereby increase sensitivity to cell cycle-dependent cytotoxicity.

## INTRODUCTION

Renal cell carcinoma (RCC) accounts for 85% of renal cancers, of which clear cell RCC (ccRCC) is the most prevalent form [1–5]. RCC responds poorly to cytotoxic drugs. Although, the prognosis for patients with this disease has recently improved due to earlier detection of RCC and expansion of treatment possibilities such as mTor inhibitors and VEGFR-targeted tyrosine kinase inhibitors [1], these treatments rarely yield complete responses. T-cell checkpoint inhibitors show promise but are not standard of care at the time of writing. Thus, RCC is still a tumor with poor clinical outcome and identification of additional cellular treatment targets is of utmost importance for improving prognosis.

CD133 is a pentaspan transmembrane glycoprotein (Prominin-1), whose specific functions are still unclear. It was initially identified as a specific marker for hematopoietic stem cells [6, 7] but more recently it has been proposed to be a stem/progenitor marker in a variety of adult tissues including normal human kidney and RCC [8–22]. However, the specificity of CD133 as a marker for stem/progenitor cells is a matter of debate (reviewed by Wu and Wu et al., [23], as it has been found on various differentiated adult epithelial cells, including renal tubular cells [10, 24–28]. Although CD133^+^ cells from several human tumors exhibit xenotransplantation potential in severe combined immunodeficiency (SCID) mice [8, 18, 19, 29], CD133^+^ cells from human RCC tissue (RCC^CD133+^cells) [27] fail to form tumors when transplanted independently in SCID mice but instead potentiate tumor engraftment when co-transplanted with CD133^−^ RCC cells [29]. Higher numbers of CD133^+^cells in RCC tissue has been correlated with increasing tumor grade [22] and was associated with a favourable prognosis in some studies [27, 30] but not others [31]. While it is unclear whether RCC^CD133+^cells are true tumor stem cells and their prognostic significance is uncertain, their ability to promote tumor formation by CD133^−^ RCC cells may contribute to the resistance of these tumors to conventional chemotherapy so that elimination of this population may improve outcomes.

Tumor necrosis factor-alpha (TNF) is a cytokine secreted by RCC with a number of tumor promoting properties [32] and inhibition or blockade of TNF has shown clinical benefit in some patients with RCC [33–35]. This seems paradoxical as TNF was first identified as a cytokine that induced hemorrhagic necrosis of experimental tumors in mice [36]. We have shown that TNF can induce both cell death and proliferation in ccRCC cells in organ culture [37], and the relative expression and ligation of TNF receptor (TNFR) 1 and TNFR2 may be a key determinant of which pathways dominate. Specifically, ligation of TNFR1 promotes cell death whereas ligation of TNFR2 more typically induces cell cycle entry. Since the low rate of proliferation of RCC^CD133+^cells may be protecting them from chemotherapy, we have investigated the effects of TNFR2 on RCC^CD133+^cells and asked whether such pre-treatment would induce proliferation and thereby render them more susceptible to cell cycle-dependent chemotherapeutic drugs [38].

## RESULTS

### Enumeration and characterization of CD133^+^cells in NK and ccRCC

**Figure 1:**
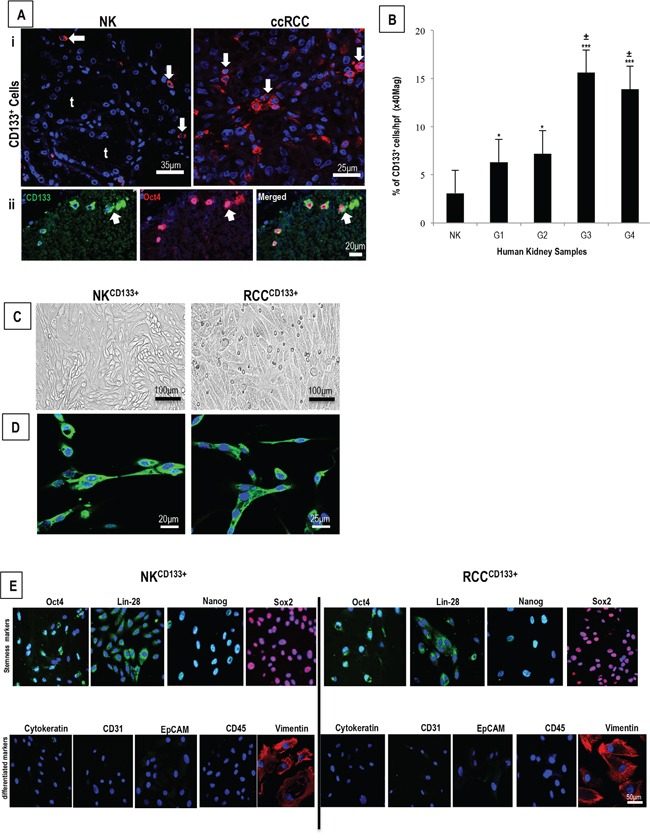
**A.** Representative confocal image of ccRCC grade 3 show a significantly larger number of CD133^+^cells as compared to NK (*arrows*) (***i***), with some CD133^+^cells also positive for stem cell transcription factor Oct4 (*arrows*) (***ii***) **B.** The increase in CD133^+^cells correlate with tumor grade (*p<0.05 - grades 1 & 2 *vs* NK; ^***^p<0.001 - grades 3 & 4 *vs* NK), with more cells detected in grade 3/4 as compared to grade 1/2 (^±^p<0.01). **C.** Phase-contrast images of tissue-enriched CD133^+^cells from NK (NK^CD133+^) and RCC (RCC^CD133+^) show spindle-shaped morphology and a scanty cytoplasm and, **D.** are strongly positive for CD133. **E.** Both the cell types show expression of stem cell markers (Oct4, Lin28, Nanog and Sox2) and vimentin but lack epithelial, endothelial and leukocyte differentiation markers (Cytokeratin, EpCAM, CD31, CD45) t-tubules; G1-G4 - tumor grades 1- 4. Error bars represent mean ± SEM, data are from at least 3 independent experiments with similar results.

Immunostaining on sections of NK demonstrated rare interstitial CD133^+^cells (NK^CD133+^) (mean <3%) within the cortex, consistent with a previous report [10]. In comparison, sections of ccRCC contained a noticeably higher frequency of RCC^CD133+^ cells (RCC^CD133+^), which increased further with tumor grade (Figure 1A *panel i*, quantified in B), ranging from mean ~6-8% in grade 1/2 tumors to 14-16% in grade 3/4 tumors. Combined-immunostaining for CD133 and a stem cell transcription factor, Oct4, demonstrated a strong nuclear staining of Oct4 in 8% of NK^CD133+^ and 12% of RCC^CD133+^cells (Figure 1A, *panel ii*). Cultures of CD133^+^cells isolated from either NK and ccRCC demonstrated spindle-shaped morphology and scanty cytoplasm (Figure 1C). Because the glycosylated form of CD133 expression that is recognized appears to vary with the antibody used for staining [39] and because different glycosylation patterns may be indicative with stemness, we tested different antibodies on our isolated cell populations. Four different anti-CD133 monoclonal antibodies (W6B3C1, AC133, ab19898, E93002) displayed concordant immunolabeling (Supplementary Figure 1) and the same cells expressed a marked signal for stem cell-associated markers [Oct4, Lin28, Nanog and Sox2) and for vimentin but lacked epithelial [cytokeratin and epithelial cell adhesion molecule (EpCAM)], endothelial [PECAM-1 (CD31)] and leukocyte [leukocyte common antigen (CD45)] markers (Figure 1E). This phenotype is most consistent with but does not absolutely establish identity as a stem cell population.

### Expression of TNF and TNFRs and responses to TNF by RCC^CD133+^ and NK^CD133+^cells

**Figure 2:**
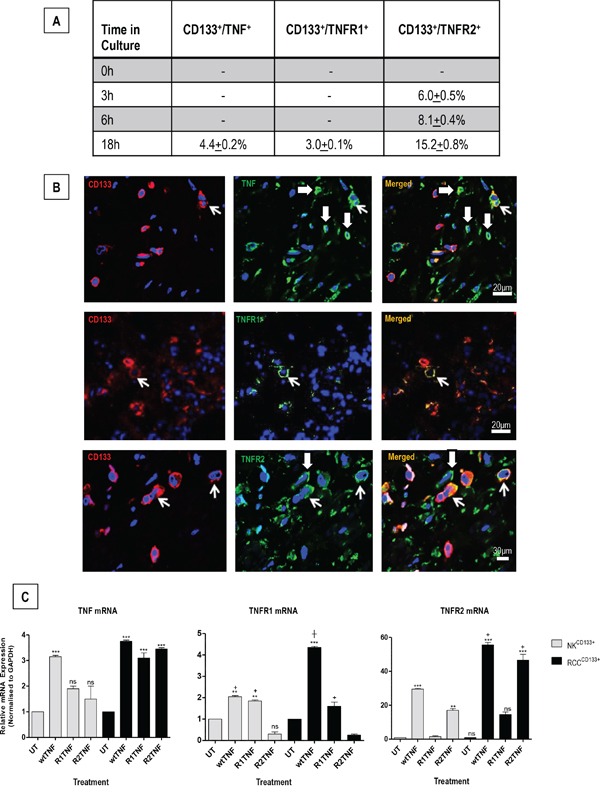
**A.** CD133^+^ cells isolated from RCC (RCC^CD133+^) were quantified for expression of TNF or TNFR1 or TNFR2 at basal level (0h) and after treatment with wtTNF at various time points (3, 6 & 18h). TNFR2 expression is detected in cells at 3, 6 and 18h, with a higher level of expression after 18h. In comparison, expression for TNF and TNFR1 is only detected after 18h cultures. **B.** Representative images of corresponding ccRCC organ cultures show staining of TNF or TNFR1 or TNFR2 in CD133^+^cells (*open arrows*), with a few CD133^−^cells also positive for TNF or TNFR2 (*shaded arrows*). **C.** qRT-PCR analysis of RCC^CD133+^ and NK^CD133+^cells at day 7 following treatment with wtTNF, R1TNF and R2TNF show mRNA expression normalized to control samples value of 1 for each cell type. Error bars represent the mean ± SEM, ^***^p<0.0001 *vs* UT; **p<0.001 *vs* UT; ^+^p<0.05 *vs* R2TNF; ^┼^p<0.05 *vs* R1TNF, ns-not significant analyzed by ANOVA and Bonferonni *post hoc*; of data from at least 3 independent experiments with similar results.

RCC^CD133+^cells were examined for protein expression of TNF and TNFRs at the basal level (untreated UT; 0h) and after treatment with wtTNF at various time points (3,6 & 18h). TNF and TNFRs were negligible in UT cultures, with TNFR2 expression detected at 3, 6 and 18h, with a higher level of expression at 18h. In comparison, TNF and TNFR1 expression were detected only in 18h cultures. (Figure 2A). Corresponding organ cultures of ccRCC demonstrated different levels of TNF (*21*+*1.1*%), TNFR1 (*6.0*+*0.5*%) and TNFR2 (*28*+*1.2*%) in CD133^+^cells. There were also a few CD133^−^ cells within the interstitial tissue that were positive for TNF (*25.0+0.2*%), TNFR1 *(2.0*+*0.1*%) or TNFR2 (*18.0+0.5%*) (Figure 2B). Treatment with wtTNF increased TNFR2 protein expression in both cultured cell types, but the effect was more pronounced in RCC^CD133+^cells with a higher expression detected at 18h (*15.2*+*0.8*%) compared to 6h (*8.1*+*0.4*%) or 3h (*6.0*+*0.5*%). wtTNF also induced TNF (*4.4*+*0.2*%) and TNFR1 protein expression (*3.0+0.1*%) at 18h but not at 6 or 3h. Interestingly, R2TNF induced TNFR2 but not TNF or TNFR1 while R1TNF induced TNFR1 but not TNF or TNFR2 (data not shown). qRT-PCR of similar cultures demonstrated induction of TNF mRNA expression by wtTNF; increases were noted but did not reach significance in NK^CD133+^cells treated with R1TNF or R2TNF compared to UT cultures (^***^p<0.0001). In comparison, all three treatments significantly induced TNF mRNA expression in RCC^CD133+^cells (^***^p<0.0001 vs UT). Notably, wtTNF and R1TNF (but not R2TNF) induced TNFR1 mRNA expression, with a more pronounced expression demonstrated in RCC^CD133+^cells and induced by wtTNF (^***^p<0.0001) than by R1TNF (^┼^p<0.05) *vs* UT cultures. TNFR2 mRNA expression was induced by wtTNF and R2TNF (not R1TNF) (^***^p<0.0001 *vs* UT cultures) with a higher level of expression in RCC^CD133+^ compared to NK^CD133+^cells (^+^p<0.05) (Figure 2C).

**Figure 3:**
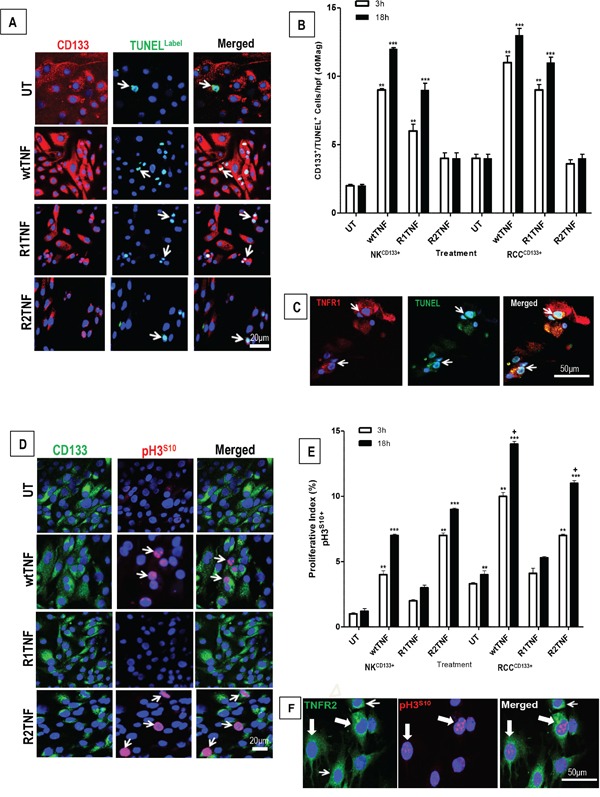
Representative confocal images of tissue-enriched CD133+cells from ccRCC and NK (RCC^CD133+^ and NK^CD133+^) immunostained for CD133 followed by TUNEL assay **A.** Untreated (UT) and R2-TNF-treated cultures show a rare signal for TUNEL (*arrows*). In contrast, cultures treated with wtTNF and R1TNF show an increased number of TUNEL^+^nuclei (*arrows*). **B.** Quantification of RCC^CD133+^/TUNEL^+^ and NK^CD133+^/TUNEL^+^ in 10 random fields of view at x40 magnification show a time-dependent increase in the number of positive cells, more pronounced in 18h than 3h cultures, with **C.** some RCC^CD133+^/TUNEL^+^cells are also positive for TNFR1 (*red*) **D.** RCC^CD133+^cells immunostained for pH3^S10^ (marker of cell cycle) show a rare pH3^S10^ positive nuclei in untreated (UT) and in R1TNF-treated cultures, while wtTNF and R2TNF-treated cultures show a strong signal (*arrows*). **E.** Percentage of RCC^CD133+^/pH3^S10+^cells in 10 random fields of view at x40 magnification, presented as proliferative index, show a time-dependent increase in proliferative cells, more pronounced in 18h cultures. **F.** Some proliferative RCC^CD133+^/pH3^S10+^cells are also positive for TNFR2 (*green*) (*shaded arrows*) with a few non-poliferative cells (pH3^S10−^) positive for TNFR2 (*open arrows*). Nuclei stained with Hoechst-33342. Bars=mean + SEM, ^***^p<0.0001; **p<0.001 ; ^+^p<0.05 signify statistically significant difference between treatments, data are from at least 3 independent experiments with similar results, ns-not significant.

We next assessed the effect of wtTNF, R1TNF and R2TNF on the induction of cell death and cell cycle activation in RCC^CD133+^ and NK^CD133+^cells. wtTNF or R1TNF each induced increased cell death in RCC^CD133+^cells as compared to UT or R2TNF-treated cultures [~27+0.9% by wtTNF and ~19+0.7% by R1TNF], positive for TUNEL in a time-dependent manner, with a marked increase in death in 18h cultures (Figure 3A and 3B and Supplementary Table 1). These effects were less pronounced in NK^CD133+^cells, in which wtTNF induced ~12+0.2% and R1TNF-induced ~6+0.2% cell death. Some RCC^CD133+^/TUNEL^+^ cells with fragmented nuclei were TNFR1^+^ (but not TNFR2^+^) with wtTNF inducing ~13+0.2% and R1TNF~6.6+0.2% (Figure 3C and Supplementary Table 2). Because R1TNF-induction of cell death in RCC organ cultures has been positively correlated with caspase activation [37], we examined similar cultures positive for TUNEL and TNFR1 for expression of cleaved caspase-3^Asp175^ by immunofluorescence (IF) and flow cytometry (FACS). 77% of the RCC^CD133+^cells treated with wtTNF that stained positive for TUNEL also stained positive for cleaved caspase 3^Asp175^ by FACS. However, only 14% of wtTNF-treated TUNEL positive NK^CD133+^cells stained positive for cleaved caspase 3^Asp175^ (Supplementary Figures 2A and 2B). These data suggest that the majority of TNF-induced cell death in RCC^CD133+^cells occurs via caspase-3 activation.

**Figure 4:**
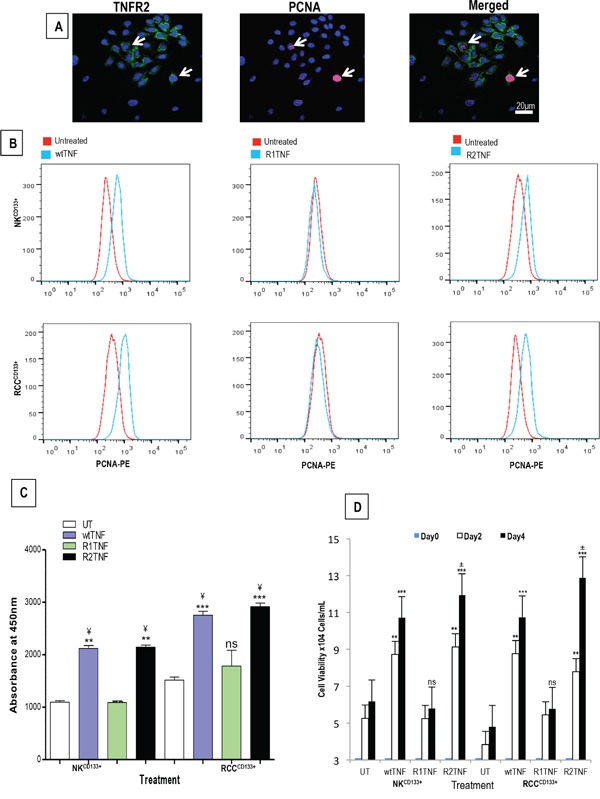
**A.** Representative confiocal images of combined staining for TNFR2 and proliferative nuclear antigen (PCNA) showing co-staining in some RCC^CD133+^ cells (*arrows*). **B.** Representative flow cytometry analysis of similar cultures from ccRCC and NK (RCC^CD133+^ and NK^CD133+^) show a high expression of PCNA induced by wtTNF- or R2TNF-treatment as compared to R1-TNF-treated and control cultures (UT) after day 4. Quantification of two cell viability assays; CCK-8 **C.** and trypan blue exclusion assay **D.** are concordant with flow cytometry showing an increase cell viability in cultures treated with wtTNF- and R2TNF as compared to UT and R1TNF-treated cultures. Error bars represent the mean + SEM, ^***^p<0.0001 *vs* UT; **p<0.001 *vs* UT, ^¥^p<0.05 *vs* R1TNF; ±p<0.05 *vs* Day 2 (R2TNF), ns-not significant; data of at least 3 independent experiments with similar results.

wtTNF and R2TNF (but not R1TNF) induced increased pH3^S10^ signal, a marker of cell proliferation, and this was more pronounced in RCC^CD133+^ cells (wtTNF ~17%, R2TNF ~13%) compared to NK^CD133+^cells (wtTNF ~7%, R2TNF ~5%) (Figures 3D and 3E). Combined-immunostaining for pH3^S10^ and TNFR2 demonstrated a strong signal for TNFR2 in some proliferating RCC^CD133+^cells (wtTNF~8% and R2TNF~6% *vs* 5% and 3% in NK^CD133+^cells)(Figure 3F). To further confirm TNF-induction of cell cycle entry, similarly treated cultures were subjected to IF with an alternative marker of cell proliferation, PCNA [40] (Figure 4A). FACS analysis of similar cultures for PCNA expression were concordant with IF findings with wtTNF and R2TNF (but not R1TNF) showing a marked expression, more pronounced in RCC^CD133+^ compared to NK^CD133+^cells (Figure 4B). Similar effects of wtTNF and R2TNF were demonstrated by cell viability assays (Figures 4C and 4D) in a time-dependent manner with increase viability in cultures at day 4 *vs* day 2 and in RCC^CD133+^ *vs* NK^CD133+^cells. These data are consistent with the interpretation that TNF induces cell death of CD133^+^ normal and malignant renal cells through TNFR1 and cell cycle entry through TNFR2 in RCC^CD133+^cells. The ability of wtTNF but neither of the muteins to increase TNF staining is unexplained but all three treatments induced TNF mRNA in isolated RCC^CD133+^cells.

### TNFR2 sensitizes RCC^CD133+^cells to killing by Cyclophosphamide (CP)

**Figure 5:**
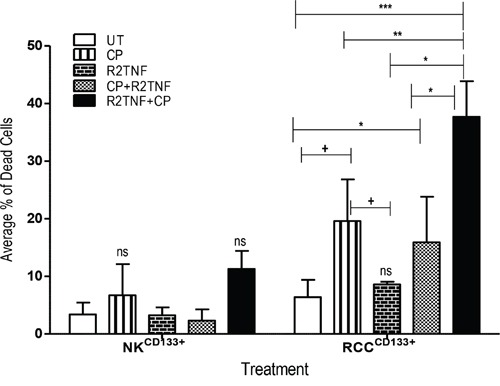
Flow cytometry analysis showing the effect of cyclophosphamide (CP) in cultures of tissue-enriched CD133^+^cells from ccRCC and NK (RCC^CD133+^ and NK^CD133+^) treated with R2TNF before and after CP or with CP alone Quantification of average percentage of dead cells stained with Annexin V and Propidium Iodide and analyzed with flow cytometry show a strong staining for both stains in RCC^CD133+^ treated with R2TNF for 48h prior to treatment with CP (1.25μM) (R2TNF+CP) for a further 48h compared to untreated controls (UT) (^***^p<0.0001) or cultures treated with CP alone (**p<0.001) or CP+R2TNF (*p<0.01). A significant increase in both staining was demonstrated in cultures exposed to CP alone *vs* UT or *vs* R2TNF alone (^+^p<0.05) but not *vs* CP+R2TNF. R2TNF+CP also induced death in NK^CD133+^ but only in 1 out of 3 experiments resulting in a no significant effect. Errors bars mean + SEM, data of least 3 independent experiments with similar results.

Having established that signling through TNFR2 does promote entry of RCC^CD133+^ into cell cycle, we next sought to determine whether R2TNF sensitizes RCC^CD133+^ or NK^CD133+^cells to killing by CP. Cells were exposed to either CP alone, R2TNF alone, or CP followed by R2TNF (CP+R2TNF) or to R2TNF followed by CP (R2TNF+CP), and then analyzed by staining with ^FITC−^-conjugated Annexin-V/Propidium Iodide (PI) and examined by FACS. In comparison with UT cultures, R2TNF+CP (^***^p<0.001), CP alone and CP+R2TNF (*p<0.05) all induced some death in RCC^CD133+^cells with the highest level of cell death induced by R2TNF+CP compared to treatment with CP alone (^+^p<0.01) or CP+R2TNF (*p<0.01). R2TNF+CP also resulted in death of NK^CD133+^ but a significant level of death was observed in only 1 out of 3 experiments, indicating that RCC^CD133+^cells are more sensitive to the cytotoxic effects of CP than are NK^CD133+^ (Figure 5). The percentage of dead RCC^CD133+^ and NK^CD133+^cells is presented in Supplementary Table 3. Moreover, representative images of ^FITC^-Annexin-V/PI and phase-contrast microscopy are shown in Supplementary Figures 3A and 3B. These data indicate that signaling through TNFR2, probably by inducing proliferation, sensitizes RCC^CD133+^cells to killing by CP.

## DISCUSSION

Our rationale for studying CD133^+^cells in NK or RCC is their potential role as renal stem cell and tumor stem cells, respectively. In the present paper, we characterized these populations both in intact tissue and in cell culture. We have evaluated their responses to TNF, as this cytokine appears to play a complex role both in NK and ccRCC, being capable of inducing both proliferation and cytotoxic responses [37]. Analyzing responses mediated by TNFR1 and TNFR2 using muteins that selectively activate one receptor or the other can separate some of the seeming contradictions in TNF responses. Our key findings are that in intact renal tissue there is a significant increase in the numbers of CD133^+^cells in RCC *vs* NK, consistent with a prior report [27]. Furthermore, the frequency of RCC^CD133+^cells increased with higher tumor grade. Both NK^CD133+^ and RCC^CD133+^cells are responsive to TNF. Specifically, ligation of TNFR2 increases transcription of TNFR2 and promotes their entry into cell cycle in organ culture. The entry of RCC^CD133+^cells into cell cycle in response to TNFR2 signaling increased susceptibility to CP-induced cell death. R2TNF also induced proliferation of NK^CD133+^cells but CP was less effective in killing them as compared to RCC^CD133+^cells.

A key question, not fully answered by our findings, is whether the interstitial CD133^+^cells in the renal cortex are or are not renal stem cells. We found that CD133^+^cells in RCC and NK were uniformly negative for the hematopoietic stem cell markers CD31 or CD45 but were strongly and persistently positive for stem cell markers (Oct4, Sox2, Nanog and Lin28) and for vimentin [41]. These data strongly suggest that they are a primitive cell type, consistent with previous studies [27, 29]. Although, we failed to detect cytokeratin in the CD133^+^ cells from both the study groups, we did however, observe its expression when driven to differentiate into an epithelial lineage (*RA-L unpublished data*), suggesting capacity of these progenitor cells to acquire markers characteristic of fully differentiated renal epithelia. Stem cell transcription factors Oct4 and/or Sox2 have been found to bind to the P1 promoter region of the CD133 gene and ectopic Oct4 or Sox2 expression has been shown to trigger the CD133P1 activity in the lung cancer cell lines [42]. CD133 marker was initially used to identify cancer stem cells (CSCs) in brain tumor [18, 19]. CD133^+^stem/progenitor cells, also referred to as tumor-initiating cells, CSCs, stem-like cells [9, 20, 23, 29, 30, 43-47], have been isolated in normal and neoplastic tissue including the human kidney and RCC [9-11, 27, 29, 45, 48, 49]. Although some CD133^+^cells have been shown to possess self-renewal properties and clonal efficiency; current literature on the use of CD133 as the only marker to identify stem cells has been questioned since its presence has been localized in a variety of adult epithelial cells including pancreas, liver and renal tubules [10, 26, 28, 50, 51] and localize to membrane protrusions [6]. In RCC, CD133^+^cells have been detected in apical membrane of tumor cells in macro/microcytic regions [27] and in perinecrotic/perivascular areas [20]. Moreover, in human kidneys, CD133 immunoreactivity was localized to proximal tubules and Bowman's capsule [50] and not restricted to stem cells. Interestingly, other groups have shown that CD133 is not only a biomarker, but also functions in cell growth, development and tumor biology [52]. Indeed, CD133 expression and post-translational modification are dynamic and reversible that is dependent on cell microenvironment and physiological regulation [53].

The tumorigenic potential of CD133^+^cells depends on the tissue of origin. For example, in human brain, CD133^+^cells are capable of self-renewal and recapitulate the original tumor when transplanted in SCID mice [19]. Similarly, CD133^+^cells from primary lung tumors show higher tumorigenic potential than their CD133^−^counterparts and are able to reproduce the original tumor in SCID mice [8]. Conversely, in RCC, undifferentiated CD133^+^cells express low level of Oct4, have a low proliferative rate [27] and fail to form tumors independently in SCID mice but support tumor growth when co-transplanted with tumor cells [29]. These reports indicate that CD133^+^cells are a heterogeneous cell population with varied function(s).

Recruitment of CD133^+^ pro-angiogenic cells in cancer has been considered to play an important role in resistance to vascular growth factor (VEGF) neutralizing antibody such as bevacizumab, possibly due to failure to inhibit their differentiation into endothelial cell types [54]. Moreover, CD133 gene polymorphism have been associated with lower overall survival rate in patients treated with bevacizumab in metastatic colorectal cancer treated [55]. These studies generally have as their premise that VEGF as a tumor angiogenic factor. Neutralization of VEGF may play an additional role in RCC as these cells, like endothelium, express VEGF receptor 2 (VEGFR2) and proliferate in response to its autocrine production. Our prior studies of malignant tubular epithelial cells in ccRCC organ culture indicated that signalling through TNFR2 induced ligand-independent transactivation of VEGFR2 and that blockade of VEGFR2 kinase activity inhibited TNFR2-induced cell cycle entry [37, 56, 57].

Tumor cells and tumor microenvironment may play a vital role in regulating self-renewal properties of CD133^+^cells in RCC [29]. Inflammation and hypoxia are characteristic features of neoplasm and facilitates tumor growth. Cancer-associated inflammation is characterized by expression of cytokines, influx of leukocytes, tissue remodeling and angiogenesis [58–60]. Cytokines have pro-tumor properties. TNF is secreted by RCC [37, 61], and increase stemness properties in RCC in 2D cultures [62]. TNF targets the quiescent/slow-cycling stem cells and promotes PI3K/AKT-driven expansion in melanoma by preventing their asymmetrical self-renewal [63]. These data strongly suggest that TNF, present in the tumor microenvironment, may influence the behaviour of CD133^+^cells in RCC and potentiate their self-renewal properties. In our preliminary studies, we have demonstrated TNF-induced upregulation of stem cell transcription genes (Oct4, Nanog and Lin28) in RCC^CD133+^cells (*RA-L, unpublished data*), consistent with a prior report by Ueda *et al* [64]. Hypoxia has been shown to influence the expression of CD133 in RCC [65], as well as in lung cancer, pancreatic cancer and glioma cells [66, 67]. We have previously reported that hypoxia both increases TNF expression and TNFR2 induction and expression of a stem cell marker Lin28 in c-kit^+^ cardiac stem cells that then enter cell cycle [68]. Collectively, these findings suggest that a transient exposure of RCC^CD133+^cells to TNF and/or hypoxia may imprint their long-lasting molecular and/or cellular changes with functional consequences.

In summary, our data confirm that CD133^+^cells are present in larger numbers in ccRCC than in NK and that they respond to TNFR2 signaling, which promotes their proliferation. While the specific role of RCC^CD133+^cells in renal tumours remain unclear, they likely have pathogenetic signficance. TNF, signaling via TNFR2, not only causes these cells to proliferate, it also increases their senstivity to cell cycle-sensitive chemotherapy. Therefore, priming these cells with a TNFR2 agonist may enhance the efficacy of chemotherapy for ccRCC.

## MATERIALS AND METHODS

### Antibodies, cytokines, and reagents

Recombinant human TNF (cat~201-TA), mouse anti-TNFR1 (cat~MAB225) and mouse anti-TNFR2 (MAB226) all from R&D Systems, Abington, UK, rabbit anti-TNFR1 (cat~ab19139) (Abcam, Cambridge, UK), rabbit anti-TNFR2 (cat~sc-7862 H202, Santa Cruz Biotechnology, Heidelberg, Germany), mouse anti-PECAM/CD31 (cat~M0823), mouse anti-cytokeratin (cat~M0821 clone MNF116) and mouse anti-CD45 (cat~M0701), mouse anti-Vimentin Clone V9 (cat~M0725) all from Dakocytomation, Ely, UK, rabbit anti-Vimentin (D21H3)(Cell Signaling Technology Inc, UK), rabbit anti-CD133 (E90032, Source Bioscience, Nottingham, UK); rabbit anti-CD133 (cat~ab19898, Abcam), mouse anti-CD133/1-W6B3C1 (cat~130-092-395) and mouse anti-CD133/1-AC133 (cat~130-090-422) both from Miltenyi Biotec Ltd, Surrey, UK, rabbit anti-human EpCAM (GTX113091, Source Bioscience); mouse anti-Sox2 (cat~MCA5660T) and mouse anti-Nanog (cat~MCA5657T, Bio-Rad Laboratories Ltd, Hertfordshire, UK), Hoechst-33342 (Thermo Fisher Scientific, Paisley, UK); rabbit anti-Lin28 (cat~ab46020), rabbit anti-Oct4 (cat~ab19857), mouse anti-phosphorylated Histone H3^S10^ (pH3^S10^) (cat~ab14955), rabbit anti-phosphorylated Histone H3^S10^ (pH3^S10^)(cat~ab5176), anti-TNF-alpha (cat~ab6671) all from Abcam, mouse anti-proliferative cell nuclear antigen (PCNA, cat~MAB424 clone PC10, Millipore, Hertfordshire, UK). Cleaved caspase-3^Asp175^ (cat~9661, Cell Signaling Technology, Leiden, The Netherlands). Tissue Dissociation Kit (cat~130-095-929), MS columns (cat~130-042-201), C-Tubes (cat~130-093-237) were from Miltenyi Biotec. TUNEL label (dUTP^−FITC^) (cat~11767291910), Terminal transferase enzyme (TdT) (cat~03333566001), deoxynuclease-1 (DNase-1) and Annexin-V-FLUOS Staining Kit all from Roche Diagnostics, Mannheim, Germany. Recombinant mutations of the TNF sequence which enable the mutated protein (“mutein”) to bind selectively to either of the TNFR subtypes; TNFR1 (R1TNF) and TNFR2 (R2TNF) [37, 40, 69]. Cyclophosphamide was from Sigma-Aldrich, St. Louis, MO.

### Tissue collection

Experiments using human tissue were performed with informed consent of patients and approval of the local ethics committee and Cambridge University hospital Tissue Bank. RCC tissue obtained from radical nephrectomy specimens was immediately excised from tumors that grossly appeared to be ccRCC. This tumor classification was later verified by routine histological assessment of paraffin-wax embedded samples. Non-clear cell histological tumor types (e.g. papillary, chromophobe, and collecting duct) [70] were excluded and only ccRCC, graded according to the four-tiered Fuhrman nuclear grading system [71] and pathologically staged based on the TNM classification [37, 72] were used. Tissue samples from 40 patients were collected and scored as Fuhrman grade 1 (n=10), Fuhrman grade 2 (n=10), Fuhrman grade 3 (n=12), and Fuhrman grade 4 (n=8). In parallel, adjacent non-tumor kidney (NK) (n=20), categorized histologically as normal kidney cortex was collected remote from the tumor site. All samples were either fixed overnight at 4°C in 4% formaldehyde in 0.1M phosphate buffer pH 7.6 and paraffin wax-embedded for immunofluorescence or snap-frozen in isopentene-cooled in liquid nitrogen. Parallel-unfixed fresh samples were processed for organ culture experiments.

### Kidney organ cultures

Organ cultures were performed as previously described [37]. In brief, duplicate 1 mm^3^ fragments of tissue from ccRCC grade 1 and NK (n=5 per study group) were immersed in M199 medium containing 10% heat-inactivated fetal calf serum (FCS), antibiotics and 2.2mM glutamine. Cultures were either left in media alone (untreated controls; UT) or treated with TNF (10ng/mL) or R1TNF or R2TNF (1μg/mL) [37, 40, 73] at various time points (0, 3, 6 and 18h). Cultures were harvested, fixed in 4% formaldehyde and embedded in paraffin-wax.

### Processing, isolation, and culture of CD133^+^cells from ccRCC and nontumor adjacent kidney (NK)

ccRCC and NK (n=10 per study group) were enzymatically digested as previously reported [68]. In brief, tissue was digested into single cell suspension using Tissue Dissociation Kit on a GentleMACs Disassociator and incubated on a MACsMix rotator (Miltenyi) for 30 min at 37°C, centrifuged at 300g for 5 min, passed through 40μm strainer to remove cell clumps. Single cell suspensions were cultured in nondifferentiating expansion medium [29] [containing 60% low-glucose Dulbecco's modified Eagle's medium-LG (DMEM-LG), 2% FCS, 40% MCDB-201, 1x insulin-transferrin-selenium, 1x linoleic acid 2-phosphate, 10-^9^ M dexamathasone, 10^4^M ascorbic acid 2-phosphate, 10 ng/mL epidermal growth factor (EFG), 10 ng/mL platelet derived growth factor, and 1000 units/mL lymphocyte inhibitory factor, 100U penicillin, 1000U streptomycin] all from Invitrogen, Paisley, United Kingdom. Enrichment of CD133^+^cells from ccRCC and NK (referred to as RCC^CD133+^ and NK^CD133+^) was achieved by magnetic cell sorting system according to the manufacturer's instructions (Mitenyi). A total of 6×10^8^ cells were magnetically labeled with CD133 microbeads and passed through the MACS column on MiniMACS Separator. The magnetically labeled CD133^+^cells were eluted from magnetic column as the positively selected cell fraction. Enriched population of RCC^CD133+^ and NK^CD133+^cells (~6×10^5^ cells/mL) were seeded in T25 flasks and transferred to eight-well glass slide chambers (Thermo-scientific, UK). Once confluent, cells were either UT or treated with wtTNF or R1TNF or R2TNF for 3 and 18h.

### Single and combined immunofluorescence (IF)

Sections of ccRCC, NK and corresponding organ cultures were immunostained for CD31 (1:50 dilution) as previously described [37, 40]. As CD133 expression has been associated with stem/progenitor cells in RCC, we co-immunostained some cultures with anti-CD133 and a panel of stemness markers (Lin28, Oct4, Nanog, Sox2) or markers for epithelial, endothelial and leukocytes (cytokeratin, CD31, EpCAM, CD45) and a mesenchymal marker (vimentin) (all used at 1:100 dilution). pH3^S10^ and PCNA (used at 1:500 and 1:200 dilution) were used to detect cell cycle activation and assessed in association with TNF and TNFRs. In addition, some cultures were subjected to co-immunostaining for TNFR1 and cleaved caspase-3^Asp175^. Antibody binding sites were detected with Northern Light-^498^ or -^557^-conjugated secondary antibodies (diluted 1:100), incubated 1h at room temperature. Hoechst-33342 (1μg/ml) was used for nuclei detection. Species-specific antiseras were used as negative controls. Slides were viewed on a Leica TCS-SPE confocal microscopy (CLSM) (Leica Microsystems, Milton Keynes, UK) and image for each fluorophore was acquired sequentially using the same constant acquisition time and settings rather than simultaneously to avoid crosstalk between channels. Images were then processed in Adobe Photoshop CS6 software.

### Determination of RCC^CD133+^ and NK^CD133+^ cell death

The effect of wtTNF, R1TNF and R2TNF treatment on cell death in RCC^CD133+^ and NK^CD133+^ was assessed using TUNEL as previously described [37, 40, 74]. Briefly, RCC^CD133+^ and NK^CD133^ cells and organ cultures from each of the four treatments were incubated with ^FITC^-dUTP-TUNEL label mix containing TdT-enzyme plus Hoechst-33342 for nuclei detection and mounted in Vectorshield mountant (Vector Laboratories, Peterborough, UK). For negative controls, TdT-enzyme was omitted and for positive controls cultures were pretreated with DNase-1 enzyme (Promega, Hampshire, UK) before TUNEL. Three experiments were performed separately. Following TUNEL, some cultures were incubated with anti-TNF and anti-TNFRs antibodies and detected using Northern Lights^568^-conjugated secondary antibody plus Hoechst-33342. In addition, cell suspensions from UT and wtTNF-treated cultures from both the study groups were labeled with an antibody to cleaved-caspase-3^Asp175^ followed by incubation with a FITC-conjugated secondary antibody and analyzed by flow cytometry (FACS) using Canto II (BD Biosciences). All slides were viewed on a CLSM.

### RNA isolation and quantitative reverse-transcription polymerase chain reaction (qRT-PCR)

Total cellular RNA was extracted from UT and wtTNF-, R1TNF-, R2TNF-treated cultures of RCC^CD133+^ and NK^CD133+^cells using the RNeasy Plus Mini Kit (Qiagen, Germany). 6μg RNA from each sample was reversely transcribed into cDNA and measured using Taqman Reverse Transcription Kit according to the manufacturer's instructions (ABI7700 System, Applied Biosystems, CA, USA). TaqMan_Gene Expression Assay ID: TNFR1 (Hs00533560), TNFR2 (Hs00153550), TNF (Hs99999043) were used. (GAPDH; Hs04420697) was used for normalization.

### Determination of RCC^CD133+^ and NK^CD133+^ cell proliferation

The effect of wtTNF, R1TNF and R2TNF on cell proliferation was evaluated by FACS using immunostaining for anti-PCNA antibody and two different cell viability assays [cell counting kit-8 (CCK-8) and trypan blue exclusion]. RCC^CD133+^ and NK^CD133+^cells serum-depleted were seeded in 12-well culture plates for FACS (1×10^5^cells/mL), for CCK-8 assay (3×10^4^cells/mL) and for trypan blue staining (5×10^3^cells/mL) in 24-well culture plates. Cultures were either left UT or treated with wtTNF, R1TNF or R2TNF for 48h and 96h. Cells were then rinsed in PBS, blocked with FcR blocking reagent (Miltenyi) and incubated with PE-conjugated antibody to PCNA- or with PE-conjugated isotype-specific antisera before examining using FACS Canto II. 10,000 events were collected per sample and the data analysed with FlowJo software. For the CCK-8, cells were treated for 48h and the WST-8 reagent (10μL/well) Sigma-Aldrich, UK was added to each well and incubated for 2h at 37°C. The absorbance optical density (OD) (proportional to the relative abundance of living cells) was measured at 450nm wavelength using a microplate reader (Bio-Rad, USA). For trypan blue exclusion assay, 200μl cells were incubated in 0.4% (w/v) trypan blue solution containing 0.81% NaCl plus 0.06% (w/v) dibasic potassium phosphate for 3 min at room temperature and counted using a haemocytometer. Viable and non-viable cells were recorded separately, and the means of 3 independent cell counts from 3 separate experiments were pooled for analysis.

### Determination of cytotoxic effect of cyclophosphamide (CP) by FACS

To evaluate the effect of CP following cell cycle activation of RCC^CD133+^ and NK^CD133+^cells by R2TNF, cells (0.5×10^5^cells/well) were seeded in 24-well plates coated with human plasma fibronectin. Cells were then either left UT or treated with CP alone or R2TNF alone, or R2TNF for 48h before CP (R2TNF+CP) (to induce cell proliferation) or after (CP+R2TNF) for a further 48h. A dose response curve was initially set up to determine the optimal effect of CP using documented concentrations (1.25, 2.5 and 5μM) [75, 76] at 24h and 48h time points. No significant difference in toxicity was demonstrated between the 3 doses, although a higher number of dead cells were evident at 48h. 1.25μM CP for 48h was therefore used for all subsequent experiments. Following treatment, cells were harvested and cytotoxic effect of CP determined by Annexin V/Propidium Iodide (PI) staining using FACS. Cells were gated as fractions of non-apoptotic live cells (Annexin V^−^/PI^−^), early apoptotic cells (Annexin V^+^/PI^−^), late apoptotic and necrotic cells (Annexin V^+^/PI^+^) and calculated as the average percentage of dead cells. FlowJo and GraphPad softwares were used to analyze the data, represented as mean ± SEM from at least 3 independent experiments.

### Data analysis

#### Cell death and proliferative indices

The average number of RCC^CD133+^ and NK^CD133+^cells positive for TUNEL were counted in 10 random fields of view at x40 magnification from each treatment divided by the total cell numbers to generate the percentage of positive cells. Similarly, the numbers of RCC^CD133+^ and NK^CD133+^cells positive for pH3^S10^ were counted and divided by the total cell numbers to generate the % of positive cells, calculated as proliferative index (PI) for each treatment. Error bars represent mean ± SEM. Each experiment was repeated at least 3 times and the same statistically significant differences between experimental groups were observed in all three independent experiments although the absolute values varied. Microsoft Excel 2013 and GraphPad Prism 5.02 softwares were used for data processing. Statistical significance was assessed by the analysis of variance test and a p-value of <0.05 were considered significant.

## SUPPLEMENTARY FIGURES AND TABLES








