# Supplementary material for: Tumor necrosis factor receptor 2-signaling in CD133-expressing cells in renal clear cell carcinoma
Source: Oncotarget. 2016 Mar 16;7(17):24111–24. doi: 10.18632/oncotarget.8125 (PMC5029688; doi:10.18632/oncotarget.8125)
Supplement: Supplementary file 1 [file oncotarget-07-24111-s001.pdf]

SUPPLEMENTARY FIGURES AND TABLES

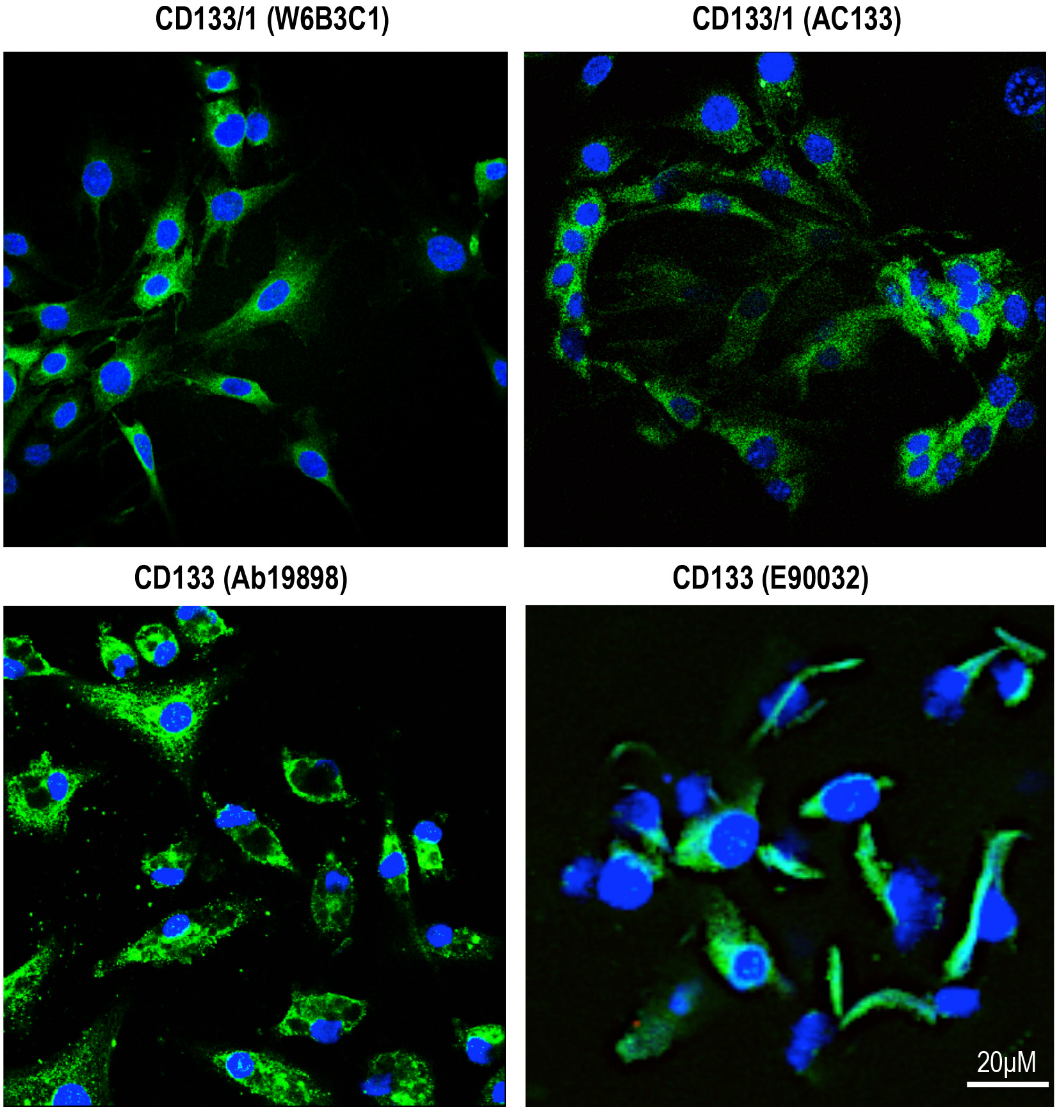

Supplementary Figure S1: Representative confocal images of RCC^{CD133+}cells stained with four different antibodies to CD133 epitope (W6B3C1, AC133, ab19898 and E90032). All the four antibodies displayed concordant immunolabeling. Magnification-x40; n=3 independent experiments with similar results.

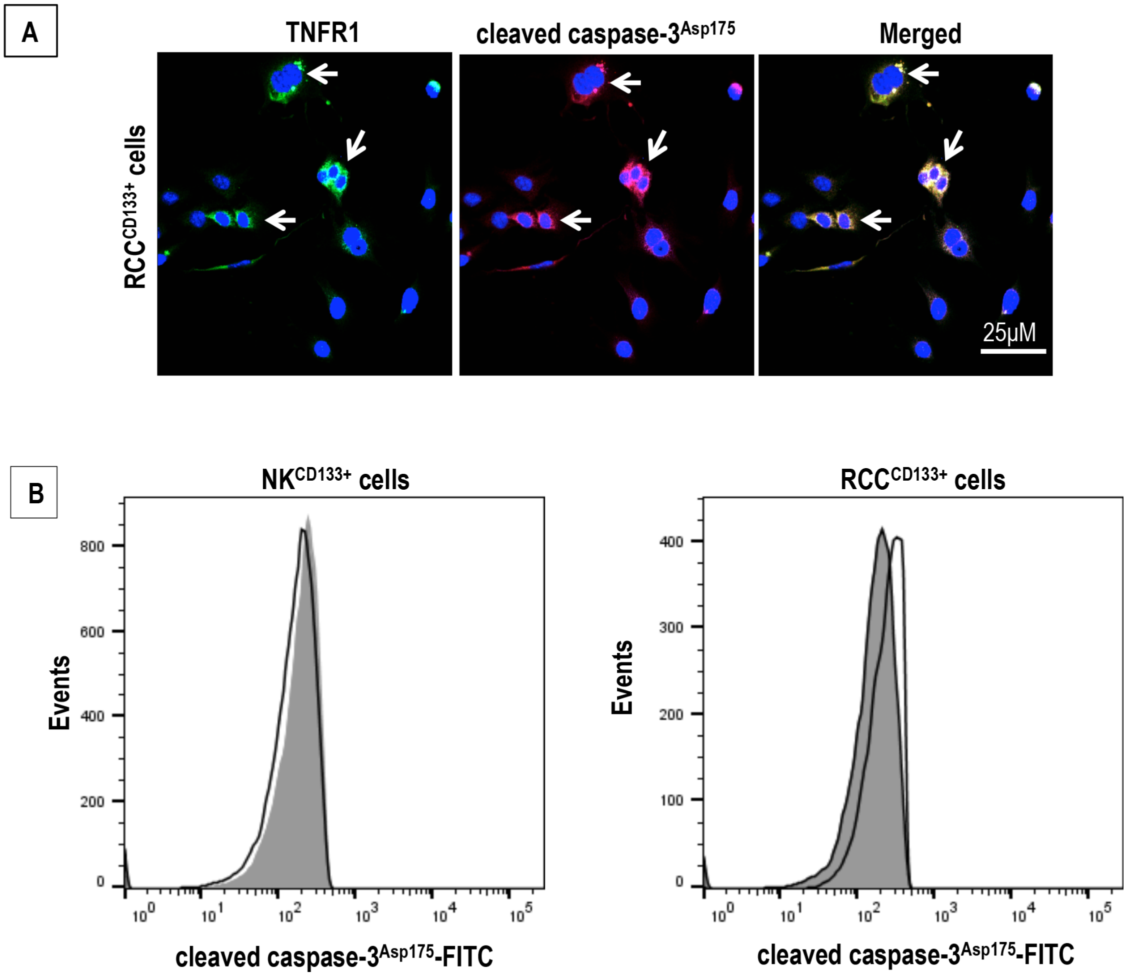

Supplementary Figure S2: A. Representative confocal images of RCC^{CD133+}cells treated with wtTNF for 18h show co-localization of TNFR1 and cleaved caspase-3^{Asp175}. **B.** Flow cytometry histograms on similar cultures of RCC^{CD133+} and NK^{CD133+}cells either untreated or treated with wtTNF show expression of cleaved caspase-3^{Asp175}, indicative of apoptotic cells in RCC^{CD133+} as compared to NK^{CD133+}cells. n=3 independent experiments with similar results.

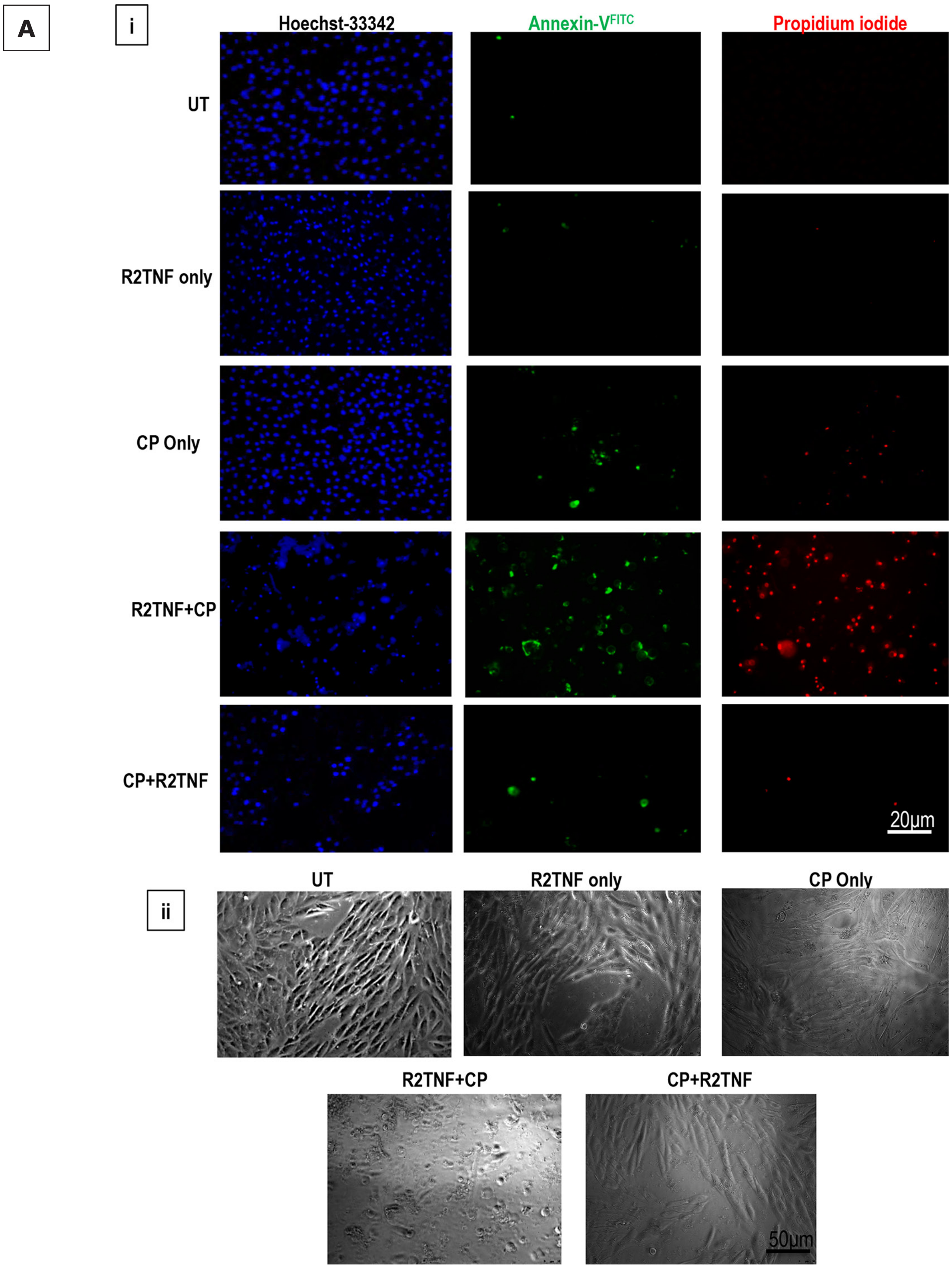
A
i
Hoechst-33342
Annexin-V^FITC
Propidium iodide
UT
R2TNF only
CP Only
R2TNF+CP
CP+R2TNF
20µm
ii
UT
R2TNF only
CP Only
R2TNF+CP
CP+R2TNF
50µm

(*Continued*)

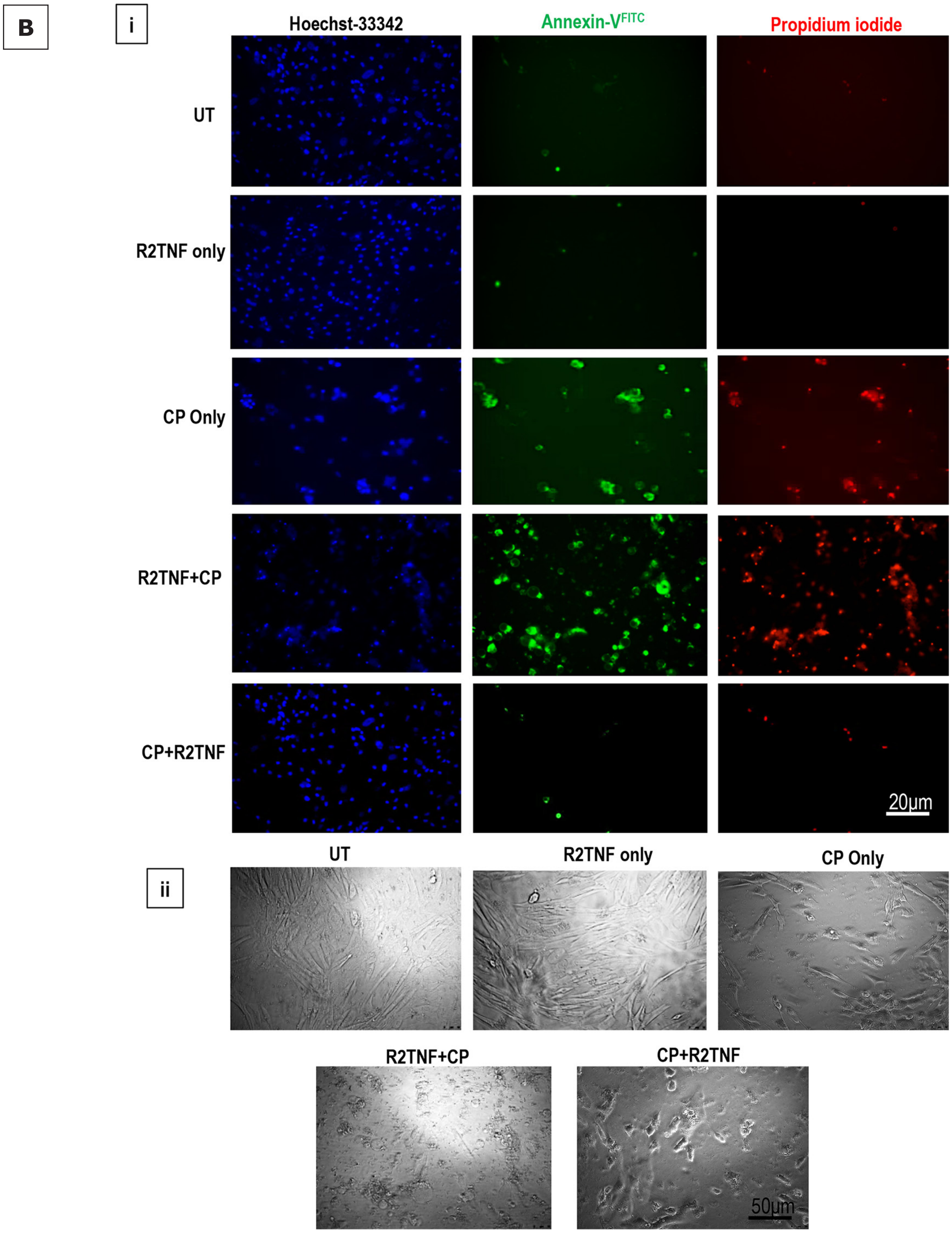

Supplementary Figure S3: Representative confocal images show cytotoxic effects of cyclophosphamide (CP) analyzed by FITC-labeled Annexin-V (*green*) and propidium iodide staining (*red*) with nuclear detected by Hoechst-33342 (blue) in A. NK^{CD133+} and B. RCC^{CD133+}cells (i). Treatment of cultures with R2TNF for 48h followed by CP (1.25µM) for a further 48h induced an increase level of cell death in both the cell types, more pronounced in RCC^{CD133+}. CP alone or CP+R2TNF also induced death but to a lesser extent than R2TNF+CP. (***ii***) Phase-contrast images of similar cultures taken using an inverted Leica DMI 4000B microscope equipped with a camera.

Supplementary Table 1: Quantification of the percentage of RCC^{CD133+} and NK^{CD133+}cells positive or negative for TUNEL in untreated (UT) and after treatment with wtTNF, R1TNF and R2TNF for 18h.

See Supplementary File: 1

Supplementary Table 2: Quantification of the percentage of RCC^{CD133+} and NK^{CD133+}cells positive for TUNEL and TNFR1 in untreated (UT) and after treatment with wtTNF, R1TNF and R2TNF.

See Supplementary File: 2

Supplementary Table 3: Quantification of the percentage of RCC^{CD133+} and NK^{CD133+}cells either left untreated (UT) or following treatment with R2TNF before and after cyclophosphamide (CP) or with CP alone.

See Supplementary File: 3
