# Supplementary material for: Tumor necrosis factor receptor 2-signaling in CD133-expressing cells in renal clear cell carcinoma
Source: Oncotarget. 2016 Mar 16;7(17):24111–24. doi: 10.18632/oncotarget.8125 (PMC5029688; doi:10.18632/oncotarget.8125)
Supplement: Supplementary file 2 [file oncotarget-07-24111-s002.docx]

| NK^CD133+^ cells | TUNEL^+^ cells | TUNEL^-^ cells |
| --- | --- | --- |
| UT | 2.0+0.1% | 98+0.1% |
| R1TNF | 6.0+0.2%^*±^ | 94+0.2% |
| R2TNF | 3.2+0.8% | 96.8+0.8% |
| wtTNF | 12.0+0.2%^***^ | 88+0.2% |
|  |  |  |
| RCC^CD133+^ cells |  |  |
| UT | 4.0+2% | 95+2% |
| R1TNF | 19.0+0.7%^**┼^ | 81+0.7% |
| R2TNF | 5.0+1.2% | 91+1.2% |
| wtTNF | 27.0+0.9%^***^ | 73+0.9% |

**Supplementary Table 1:** Quantification of the percentage of RCC^CD133+^ and NK^CD133+^ cells positive or negative for TUNEL in untreated (UT) and after treatment with wtTNF, R1TNF and R2TNF for 18h.

^***^p<0.001 vs UT; ^**^p<0.01 vs UT; ^*^p<0.05 vs UT; ^±^p<0.005 vs wtTNF; ^┼^p<0.05 vs wtTNF. Similar results were observed in at least 3 independent experiments. P values represent mean ± SEM.
