# Supplementary material for: Tumor necrosis factor receptor 2-signaling in CD133-expressing cells in renal clear cell carcinoma
Source: Oncotarget. 2016 Mar 16;7(17):24111–24. doi: 10.18632/oncotarget.8125 (PMC5029688; doi:10.18632/oncotarget.8125)
Supplement: Supplementary file 3 [file oncotarget-07-24111-s003.docx]

| NK^CD133+^ cells | TUNEL^+^/TNFR1^+^ |
| --- | --- |
| UT | 1.2+0.1% |
| R1TNF | 4.2+0.2%^*±^ |
| R2TNF | 1.5+0.1% |
| wtTNF | 7.0+0.3%^***^ |
|  |  |
| RCC^CD133+^ cells |  |
| UT | 2.0+0.1% |
| R1TNF | 6.6+0.2%^*┼^ |
| R2TNF | 2.5+0.2% |
| wtTNF | 13.0+0.2%^***^ |

**Supplementary Table 2:** Quantification of the percentage of RCC^CD133+^ and NK^CD133+^ cells positive for TUNEL and TNFR1 in untreated (UT) and after treatment with wtTNF, R1TNF and R2TNF.

^***^p<0.001 vs UT; ^*^p<0.05 vs UT; ^±^p<0.05 vs wtTNF; ^┼^p<0.005 vs wtTNF. Similar results were observed in at least 3 independent experiments. P values represent mean ± SEM.
