# Supplementary material for: Tumor necrosis factor receptor 2-signaling in CD133-expressing cells in renal clear cell carcinoma
Source: Oncotarget. 2016 Mar 16;7(17):24111–24. doi: 10.18632/oncotarget.8125 (PMC5029688; doi:10.18632/oncotarget.8125)
Supplement: Supplementary file 4 [file oncotarget-07-24111-s004.docx]

**Supplementary Table 3**: Quantification of the percentage of RCC^CD133+^ and NK^CD133+^ cells either left untreated (UT) or following treatment with R2TNF before and after cyclophosphamide (CP) or with CP alone.

|  | **NK^CD133+^**  **(dead cells)** | **RCC^CD133+^**  **(dead cells)** |
| --- | --- | --- |
| **UT** | 5.46+1.33% | 9.40+3.36 |
| **CP** | 6.13+1.32% | 20.13+38.18^*┼^ |
| **R2TNF** | 4.63+1.86% | 9.13+9.07 |
| **CP+R2TNF** | 4.30+0.35% | 15.80+7.99^*±^ |
| **R2TNF+CP** | 11.43+8.15% | 40.33+31.50^***^ |

^***^p<0.001-R2TNF+CP *vs* UT; ^*^p<0.05-CP or CP+R2TNF+CP *vs* UT;

^┼^p<0.01-CP *vs* R2TNF+CP; ^±^p<0.01-CP+R2TNF *vs* R2TNF+CP. Similar results were observed in at least n=3 independent experiments. P values represent mean ± SEM.
